# Current and Future Perspectives of Bioactive Glasses as Injectable Material

**DOI:** 10.3390/nano14141196

**Published:** 2024-07-13

**Authors:** Andreea-Luiza Mîrț, Denisa Ficai, Ovidiu-Cristian Oprea, Gabriel Vasilievici, Anton Ficai

**Affiliations:** 1 Science and Engineering of Oxide Materials and Nanomaterials, Faculty of Chemical Engineering and Biotechnologies, National University of Science and Technology Politehnica Bucharest, Gh. Polizu 1–7, 011061 Bucharest, Romania; luiza.mirt@icechim.ro; 2 National Center for Scientific Research for Food Safety, National University of Science and Technology Politehnica Bucharest, Splaiul Independentei 313, 060042 Bucharest, Romania; denisa.ficai@upb.ro (D.F.); ovidiu.oprea@upb.ro (O.-C.O.); 3 National Center for Micro and Nanomaterials, National University of Science and Technology Politehnica Bucharest, Splaiul Independentei 313, 060042 Bucharest, Romania; 4 National Institute for Research & Development in Chemistry and Petrochemistry—ICECHIM, 202 Splaiul Independentei, 060021 Bucharest, Romania; gvasilievici@icechim.ro; 5 Department of Inorganic Chemistry, Physical Chemistry and Electrochemistry, Faculty of Chemical Engineering and Biotechnologies, National University of Science and Technology Politehnica Bucharest, Gh. Polizu 1–7, 011061 Bucharest, Romania; 6 Academy of Romanian Scientists, Ilfov Street 3, 050044 Bucharest, Romania

**Keywords:** biomaterials, bioactive glass, hydrogel, cements

## Abstract

This review covers recent compositions of bioactive glass, with a specific emphasis on both inorganic and organic materials commonly utilized as matrices for injectable materials. The major objective is to highlight the predominant bioactive glass formulations and their clinical applications in the biomedical field. Previous studies have highlighted the growing interest among researchers in bioactive glasses, acknowledging their potential to yield promising outcomes in this field. As a result of this increased interest, investigations into bioactive glass have prompted the creation of composite materials and, notably, the development of injectable composites as a minimally invasive method for administering the material within the human body. Injectable materials have emerged as a promising avenue to mitigate various challenges. They offer several advantages, including minimizing invasive surgical procedures, reducing patient discomfort, lowering the risk of postoperative infection and decreasing treatment expenses. Additionally, injectable materials facilitate uniform distribution, allowing for the filling of defects of any shape.

## 1. Introduction

Biomaterials play an essential role in today’s world, facilitating the healing of injured tissues and restoring vital functions. Metals, ceramics, glass, plastics, and even living cells and tissues can be used to fabricate biomaterials aimed at supporting, enhancing or replacing damaged tissues [1]. The first definition was given in 1976 by the European Society for Biomaterials. Since then, it has changed due to the evolution of the field; biomaterial is now defined as “a material designed to interact with biological systems to assess, treat, enhance or replace any tissue, organ or function of the body [2].

Biomaterials have traditionally been used to replace damaged or unhealthy tissues. The production of the first biomaterials was carried out with the aim of obtaining the most bioinert materials possible and thereby minimizing the appearance of scar tissue at the interface with the host tissues [3]. The year 1969 marked the development of bioactive glass by Professor Hench, who developed a new biocompatible substance that could be combined with calcium to heal broken bones utilizing silica (glass) as the primary material. This imitates healthy bone and encourages the formation of fresh new bone when introduced into the bone tissue [4]. Its ability to regenerate and repair tissues in need led to the emergence of a third generation of biomaterials—bioglass [5].

Bioactive glasses (BG) are being broadly considered for bone tissue engineering applications based on their ability to bind strongly to bone, which is mediated by the formation of a carbonated hydroxyapatite surface layer [6]. Third-generation bioactive glass, composites, macroporous foams, injectable materials and others are designed to trigger genes that promote regeneration of living tissues [7,8].

The enormous development of engineered materials has stimulated the extension of injectable therapy to biomaterials, allowing several traditional treatments to become less invasive. Injectable biomaterials have a number of advantages, such as the controllable release of therapeutic agents, structural and physical framework, or stroma and stimulation of biological events. By combining them with cells, genes or growth factors, the results obtained will be enhanced, creating loading materials that can be encapsulated, transported, retained or delivered in order to regenerate and repair tissues [9]. One drawback of injectable biomaterials is that their study becomes more superficial as new materials, new applications of known materials, and new combinations of materials emerge [10].

Injectable biomaterials are materials that can be managed and processed at room temperature or at ambient temperature, with the ability to be extruded using a syringe and then injected into the target site, where they later solidify in response to body conditions to mold into the shape of the defect [11,12]. Injectability is commonly defined as either the force required to inject a material or the percentage by weight of extruded material relative to the original material under given conditions [11]. According to their characteristics, injectable materials can be divided into injectable hydrogels, which are polymers containing hydrophilic chains/segments that are water-soluble, and hydrophobic materials, which contain mainly hydrophobic chains or moieties [12].

From a clinical perspective, the use of injectable materials has many advantages, as it minimizes patient discomfort and the risk of infection, minimizes the duration of intervention, accelerates postoperative recovery, and reduces treatment costs and scar formation [13]. Another benefit of these materials is that they can be prepared even before application, are simple to prepare and can easily fill irregular-shaped defects [7]. However, there are also limitations, especially those of a mechanical nature; these injectable components can even flow from the intended site because the injectable material must be fluid, which limits the rigidity and leads to injectable agents that are too “soft” to fully match the local tissue [14]. Also, in the case of defects occurring near blood vessels, there is a possibility of causing strokes if the injected material has low viscosity [15].

The continued development of new injectable biomaterials shows that there is a strong tendency, due to clinical needs, for more advanced medical therapies and diagnostics [9]. Injectable biomaterials are versatile in nature, with a multitude of applications in tissue engineering involving bone or cartilage replacement, musculoskeletal conditions, cardiovascular tissues, bone cement, bone defect fillers and skin [10]. They offer an alternative for medical emergencies requiring bone reconstruction, and surgical intervention has more disadvantages than advantages [16].

The aim of this research is to explore how bioactive glasses, when incorporated as cement or hydrogels, can be applied in the field of biomedical engineering. By analyzing their unique properties, the goal is to distinguish the advantages and disadvantages associated with these distinct forms of integration. In essence, the question asked is how bioactive glasses can be used in combination with inorganic or organic matrix to formulate injectable materials that can overcome specific challenges and make progress in biomedical applications.

## 2. Cements

Bone cement is an injectable and self-setting material that has recently become widely used in hard tissue repairs, such as in orthopedics, orthodontics and plastic surgery [9]. Once cross-linked, the cement has a compact network structure or a degree of crystallization that has a relatively higher elastic modulus than hydrogels [12]. Bone cement acts as a binding material, connecting two substances. It can be characterized as a blend of powder and liquid components, solidifying upon mixing and securely adhering to the body upon implantation. This definition underscores its unique flexibility, allowing it to effectively stabilize and fix materials at fractured sites within the living body as required [17,18,19].

Once implanted, the cement should ideally exhibit bioactivity and bioresorbability while providing the necessary mechanical support to distribute the load with the adjacent bone tissue [20,21]. For cement to be ideal for injection into bone defects, it should meet the following conditions:

- Be easily injectable with appropriate homogeneity, cohesion and viscosity;
- Have an adequate curing time;
- Have a low risk of inducing necrosis;
- Have proper tensile, compressive and shear strength in accordance with the injection site;
- Have stiffness after curing similar to that of the surrounding bone;
- Have a high radiopacity to be easily distinguishable from surrounding tissues on x-ray imaging;
- Be bioactive;
- Have a resorption rate similar to that of the new tissue formation, such as during this complex process of resorption and new bone formation, the characteristics to be maintained properly;
- Have micro- and macropores to allow nutrient transfer, angiogenesis and osseointegration to occur [20].

Some of the most important aspects to be considered are the rheological properties of a bone replacement paste, represented by injectability, cohesiveness and viscosity. Injectability can often be improved by using a higher liquid-to-powder ratio in the formation of the paste, which results in lower viscosity. However, this often leads to longer curing times, higher solid–liquid segregation tendency and lower ultimate strengths. Also, when a paste, which is a two-phase mixture of a finely divided solid and a liquid, is subjected to a pressure gradient, the liquid may flow faster than the solid, leading to local changes in the composition of the paste [22,23]. What makes injectable cements increasingly used are their advantageous characteristics, such as their plasticity and formability, as well as in situ self-healing and implantation through non-invasive procedures [22].

**Figure 1:**
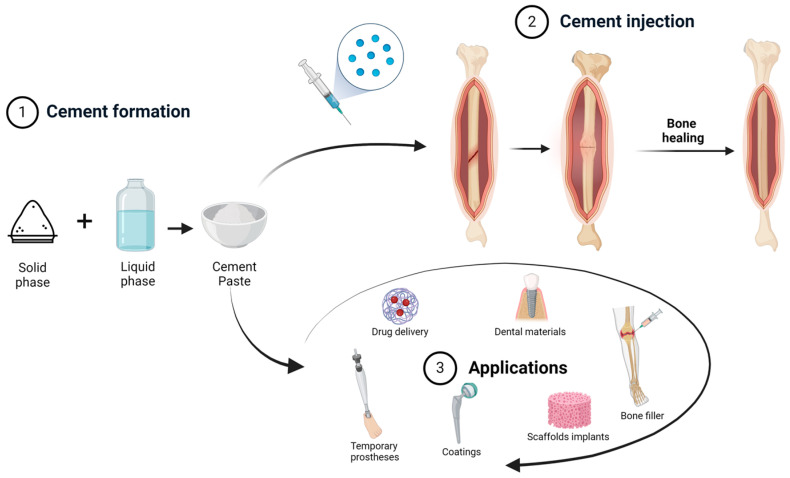
Cement paste formation and applications [24].

Depending on their chemical composition, injectable bone cements can be classified as acrylic bone cements (ABCs), calcium phosphate cements (CPCs), calcium sulfate cements (CSC) and filamentary composite materials. Alternatively, depending on the nature of the orthopedic application, injectable cements may be grouped into those for high-load-bearing applications (ABCs), medium load-bearing applications (ABCs, several CPC and certain CSC) and low-load applications (certain CPCs and CSC) [23] (Figure 1).

### 2.1. Acrylic Bone Cements (ABCs)

Acrylic bone cement is a dominant fixation material that is used in orthopedics, especially in joint arthroplasty, and this category includes poly(methylmethacrylate) (PMMA) polymer. Originally, this polymer emerged in the 1928s under the name Plexiglass as a replacement for transparent glass, and since 1960, it has been used in biomedical applications, particularly as a method of fixing joint replacements to the bone [25]. For more than 50 years, ABCs, in particular, PMMA-based formulations, have been widely used as an anchoring/fixing agent in implants such as hip implants, knee implants and a wide variety of other arthroplasty fixation.

PMMA is prepared by mixing two components, a liquid phase and a powder phase [26]:

- The liquid component contains three basic ingredients: MMA monomer; N,N-Dimethyl-p-toluidine as an accelerator of the polymerization reaction; and hydroquinone, which acts as an inhibitor, preventing premature polymerization of the monomer. This is a volatile, transparent, low-viscosity component.
- The solid component also contains three basic ingredients: PMMA granules; benzoyl peroxide, which acts as an initiator; and barium sulfate or zirconium dioxide, which is added to obtain radiopacity.

Following the success of ABCs in arthroplasty fixation, injectable ABCs have emerged with applications in stabilizing vertebral compression fractures or straightening osteoporotic vertebral body fractures, namely vertebroplasty and kyphoplasty. In this case, injectable ABCs contain an additional amount of radiopacifier. In addition to these applications, ABCs mixed with bioglass have also been investigated as drug delivery [27], orthodontic adhesive [28] or coating material [29], all demonstrating non-cytotoxicity, low cost and minimal inflammatory reactions with the host tissue.

### 2.2. Calcium Sulphate Cements

Calcium sulfate has a history of over 90 years of use in orthopedics as bone void fillers or in the delivery of various other bioactive molecules [30]. Its versatile character is due to its fast and complete in vivo resorption, uniform crystal structure and very few trace elements in its composition [31].

Calcium sulfate, or “gypsum”, is a mineral that occurs naturally as calcium sulfate dihydrate (CaSO_4_·2H_2_O) [32]. Before its use in the medical field, it undergoes purification to lose naturally occurring impurities such as silicates, lead and strontium. The first step is water removal by calcination at 110 °C, resulting in calcium sulfate hemihydrate. The hemihydrate obtained in the presence of water leads to a recrystallization of the hemihydrate into a dihydrate by a slightly exothermic process [33,34].

When calcium sulfate dissolves, it produces an acidic environment (pH = 5.6) that helps to restrain bacterial activity. Its reabsorption takes place in 6–8 weeks with a minimal increase in serum calcium levels and, therefore, leaves the hydroxyapatite particles as a scaffold to promote bone growth [34]. However, its rapid resorption can be a problem as it may be resorbed before the bone tissue is replaced by hydroxyapatite. For this reason, in many cases, a biphasic material is used, whereas the calcium sulfate phase is resorbed, pores are created in the material where cells and bone tissue can penetrate and accelerate the resorption of the other phase, leading to new bone formation [35].

It has been shown that when bioactive glass is mixed with CPCs, injectable bone cements with slower resorption and better osteogenic performance are developed. However, the content of bioactive glass used is very low as it increases the pH in contact with body fluid, which leads to alterations in the hydration reactions of calcium sulfate, resulting in its incomplete hardening. Consequently, this mixture improves the biogenic property but deteriorates the long-term mechanical support [18]. The current literature supports the use of calcium sulfate in several clinical applications, including filling for bone defects [31,36,37], chronic osteomyelitis [38,39], furcation and periodontal defects [40] and drug release [39].

### 2.3. Calcium Phosphate Cements

The concept of calcium phosphates in medical applications first emerged in the early 1980s due to clinical studies on humans and animals [40]; since then, it has been intensively studied, either biologically, morphologically or structurally [41,42,43]. Its use as a bone substitute resulted from its chemical similarity to the mineral phase of natural bone, which is biocompatible, osteoconductive and resorbable [44,45]. However, their slow degradation rate, low strength and the absence of macroporosity for bone ingrowth can be an impediment for certain applications [46,47].

Bone repair by conventional calcium phosphates therapies can be achieved in different forms, including implantation of bone grafts either as blocks or as granules (it is necessary to have prior knowledge about the shape and size of the defect, implantation of scaffolds by 3D modeling of the substitutes), and there is a possibility that the scaffold is not fully adapted to the shape of the defect [48,49,50]. These limitations can be surmounted by the production of calcium phosphate injectable materials, with the great advantage of injecting it as a paste that can be easily shaped and used through minimally invasive clinical procedures [49].

Calcium phosphates are generally synthesized by wet chemical methods such as sol–gel synthesis and precipitation, hydrothermal synthesis or dry/solid-state reactions. Regardless of the method of preparation, the products obtained have a limited range of specific compositions and morphology [50]. CPCs consist of a liquid phase and at least one calcium phosphate precursor that can be injected into the defect area [51]. In contact with aqueous media, self-induced dissolution–precipitation reactions occur with the formation of calcium-deficient hydroxyapatite [52]. The processing time of these cements is limited as a result of the ongoing curing reaction [53].

CPCs provide bone biocompatibility; however, these materials should ideally degrade at the same rate as the new bone formation, which leads to continuous support during the healing. Due to low intrinsic porosity, CPCs do not provide tissue growth in conjunction with degradation [54]. Therefore, degradable polymer microspheres have been introduced into the ceramic matrix, which has been demonstrated to increase the degradation rate and facilitate bone ingrowth [55,56,57].

In obtaining a paste similar to bone structure, the mechanical properties must be taken into account. Although CPCs have low mechanical characteristics, they still resemble cancellous bone [56]. A promising strategy to improve strength and fracture resistance is the reinforcement of calcium phosphate with fibrous materials, like polymers or oxides, such as ZrO_2_ or Al_2_O_3_; these biocomposites provide mechanical strength [51].

The most important applications of CPCs are in bone tissue engineering in the form of pre-stable scaffolds or injectable pastes to deliver stem cells, drugs and growth factors. The incorporation of bioactive molecules, cells, polymers and bioactive glass enhances the properties of CPCs—promoting bone regeneration and broadening their clinical applications [57].

## 3. Hydrogels

Hydrogels are three-dimensional hydrophilic, physically or chemically bonded polymer networks that can act as a barrier to preventing bacterial infection and creating a suitable environment for tissue regeneration [58]. In other words, hydrogels are hydrophilic gels or colloidal gels in which water is the dispersion medium; its amount varies according to different factors and is provided by capillary, osmotic and hydration forces [59]. Another characteristic of hydrogels is their ability to retain specific compounds that do not change the structure of their swollen state when exposed to specific pressures [60]. Also, they can significantly enhance bone vascularization and the impeachment of mineralized collagen [61].

The process that leads to the formation of a hydrogel is called gelation, which is based on intermolecular reactions between segments of a linear polymer. The molecular weight increases to a point where it becomes infinite, as does the relaxation time, which is called the gelation point, at which point there is no flow in the system, and the cross-linked material becomes homogeneously distributed in the recipient. Research is currently aimed at developing injectable hydrogels, which undergo in situ gelation by thermal stimulation. They can be administered in liquid form, gelling at body temperature, as an alternative to complex surgical procedures [62,63]. The advantage of hydrogels over scaffolds is that they can fill small and irregular defects and incorporate cells exerting less stress over them and therapeutic agents, which provides a new opportunity to regenerate and repair damaged tissues [64,65].

Regarding the formation of an injectable product, the gelling process is an essential step, so it is important to select a hydrogel preparation method that is consistent with the structure and intended application. There are two main categories of hydrogels depending on the cross-linking mechanism: physically cross-linked gels and chemically cross-linked gels, which are differentiated by the formation of covalent bonds [64]. The degree of cross-linking influences the swelling properties and integrity of the hydrogels. As the degree of cross-linking increases, the hydrogel becomes less flexible in shrinking, swelling or phase change in response to stimuli [65].

Physical hydrogels are semi-solid systems in which the polymeric assemblies are formed by non-covalent bonds that have low energy and a finite lifetime, such as hydrophobic interactions, ionic interaction, hydrogen bonds, supramolecular chemistry, charge condensation and crystal formation [64]. They exhibit relatively mild preparation conditions, which are favorable for medical applications, and reversible sol–gel conversion because very low energies are required to break down the physical interactions between molecules.

**Figure 2:**
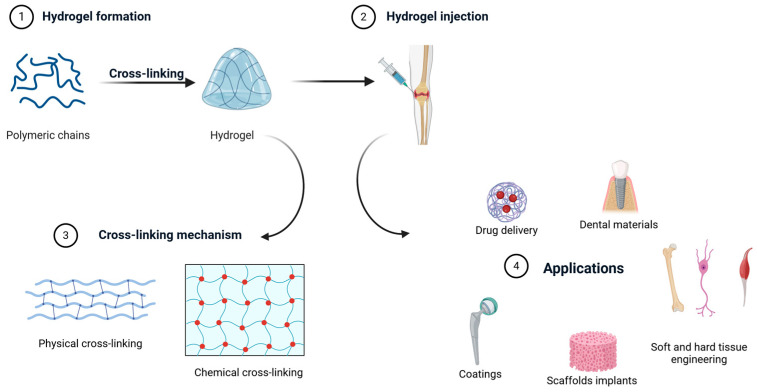
Hydrogel formation and applications [24].

The covalent bonds that form between polymer chains are the basis for the formation of chemical hydrogels, and this cross-linking is irreversible. This type of hydrogel is a macromolecule with an infinite relaxation time, which is a stable, adjustable structure with good mechanical properties. Chemical cross-linking occurs under certain special conditions and depends on the formation of covalent bonds between reactive groups grafted onto the polymerization backbone [64]. These conditions include various processes such as Michael addition [66], click chemistry [67], enzyme-mediated cross-linking [68] and photopolymerization [69] (Figure 2).

Natural materials and biocompatible synthetic polymers have been explored to fabricate injectable bioglass-based composite hydrogels for tissue engineering. Fibrous and nanofibrous materials are used because they resemble the extracellular matrix [70]. Common natural polymers used in hydrogel formulations that contain bioglass are presented below. Combining inorganic and organic matrices is crucial for bone tissue repair, allowing customization to achieve specific properties. The inorganic matrix contributes to bioactivity and mechanical strength, whereas the organic matrix provides resilience and shape formability [71].

### 3.1. Alginate

Alginate (ALG) is a natural, intensively studied polysaccharide that has demonstrated great benefits as a biomaterial in biomedical applications such as cell delivery, scaffold formation or treatment of organ failure [72,73]. It is considered to be biocompatible, non-toxic, biodegradable and non-immunogenic, and it is found in abundance and has a low price [74,75]. ALG is found in seaweed and is commonly extracted from brown algae (*Laminaria hyperborea*, *Laminaria japonica*, *Laminaria digitata*, *Ascophyllum nodosum*, *Macrocystis pyriferath*) by treatment with aqueous alkaline solutions [74]. This linear polysaccharide is composed of two copolymers, α-L-guluronic acid and β-D-mannuronic acid, which provide characteristic strength and flexibility [75]. Depending on the extraction source, alginate contains different percentages of mannuronic and guluronic acid, which influence the material properties [76]; for example, the mechanical properties of alginate depend on the ratio of these repeating units together with their molecular weight [77].

Alginate hydrogel can be formed by various approaches, such as ionic and covalent cross-linking [78]. In the presence of divalent ions, such as calcium ions, alginates have the ability to form gels by cross-linking through the carboxyl groups in the polymer chains. Under pregel circumstances, they are ionically complexed with positively charged polyelectrolytes; in this manner, they form an alginate-based system [79].

Ionic cross-linking occurs with the help of divalent cations, which interact with guluronic acid to form tightly bound ionic bridges [80]. Mannuronic acid monomers form weak junctions with divalent cations by the coordination of divalent ions with four-carboxyl groups, resulting in an egg-box arrangement. This cross-linking has two possibilities for obtaining alginate hydrogels by internal or external gelation. Covalent cross-linking of hydrogels generates strong and irreversible chemical bonds and is a promising way to achieve controlled cross-linking of the mechanical properties of alginate-based hydrogels. However, this method is not suitable for in situ scaffold injection, with possible exceptions being photoreticulable and viscoelastic hydrogels with shear-thinning behavior [81].

The applications of alginate are manifold [82], and it can be easily modified into any form, such as hydrogels, microspheres or microcapsules, sponges, foams, tubes and fibers. Its cytocompatibility, water solubility, ability to undergo in situ gelation, and prolonged release of active agents have made alginate intensively studied in bone regeneration [83,84,85].

### 3.2. Chitosan

Chitin is the second most abundant natural polysaccharide on the planet, originating from the outer shells of crustaceans in insect exoskeletons as well as in fungal cell walls [84]. Chitin has great potential in wound healing, with current research focusing on its biomedical applications [83]. Chitosan is obtained by alkaline deacetylation of chitin, and it has a linear structure consisting of randomly D-glucosamine and N-acetylglucosamine units, which are linked by β(1-4) glycosidic bonds [85,86,87].

Chitosan is degraded in the body by lysozyme—a non-specific protease, into non-toxic oligosaccharides by depolymerization of β-1,4-glycosidic bonds and deacetylation of N-acetyl bonds [88,89]. Its solubility is influenced by the positive charge of amino groups, and its homeostatic activity is favored by its positive charge, thus being able to bond with the negative charge of blood cell membranes [88]. The biocompatibility of chitosan decreases with the increase in deacetylation, solubility and degradation rate [3]. Given its similarity to glycosaminoglycans—the main components of the extracellular matrix of bone and cartilage—chitosan can improve cell adhesion and proliferation [90,91,92].

Since this polymer is known to protect osteoblast proliferation and has antibacterial and antifungal properties, chitosan is often found in porous scaffolds and wound healing [93,94]. Owing to their controllable level of drug release, cross-linked chitosan hydrogel matrices are found to be attractive in drug delivery applications [83]. Chitosan and β-glycerophosphate-based thermogels are used as injectable osteogenic agents due to their viscosity and gelation time [93].

Yao et al. [94] demonstrated that combinations of BG and chitosan can impact the mechanical properties as well as their wet extensibility. Also, the incorporation of bioglass to chitosan composites can enhance protein adsorption due to an increase in the number of protein binding sites, leading to better cell adhesion. Bioactive glass nanoparticles also provided a more intense effect than micro-sized bioglass. The incorporation of bioactive glass particles into chitosan-based hydrogels has certain limitations, such as the chitosan dissolving too quickly, causing poor mechanical properties [95]. One way to overcome this limitation may be to modify chitosan through partial quaternization [96] or to incorporate the bioglass as microbeads [90].

### 3.3. Collagen

Collagen is among the most abundant biomaterials and the principal organic component of bone tissue, with a diverse range of biomedical applications [97,98,99,100,101]. It can be processed in various forms, from sheets and sponges to scaffolds and injectable materials [100]. It is found in the composition of natural bone, which is made up of collagen fibrils that are mineralized by hydroxyapatite-like phases, specifically calcium phosphate phases [102]. In most tissues, it forms a structural network due to its high degree of polymorphism [103,104].

Collagen is composed of three polypeptide strands arranged in a left-handed, polyproline II-type (PPII) helical conformation, coiling around each other with a one-residue stagger to form a right-handed triple helix. The close arrangement of PPII helices within the triple helix necessitates that every third residue is Glycine (Gly), leading to a recurring XaaYaaGly sequence, where Xaa and Yaa represent any amino acid [103].

The properties of collagen, especially type I collagen, are similar to those found in human tissue, such as its low immunological reaction index and its depth. Despite this, due to the low mechanical strength, the use of collagen is limited; creating crosslinks in the material may be a strategy used to overcome this limitation [104]. In addition, to stimulate osteoinduction, collagen can be incorporated with bone morphogenetic proteins, giving mixed results [105].

Collagen hydrogels are rapidly absorbed in vivo and are difficult to mineralize into solutions that mimic human fluid [106,107]. The incorporation of inorganic materials such as CPCs and bioactive glass particles has been shown to increase mineralization and bioactivity but also to improve mechanical properties [108,109].

### 3.4. Gelatin

Gelatin is a natural, nontoxic, colorless protein derived from the denaturation of collagen by hydrolysis [108]. It has a tissue repair capacity due to its precursor-like structure that shows a high similarity to the extracellular matrix, stimulating cell adhesion [90]. Compared to collagen, gelatine can absorb up to 100 times more water, increasing cell spreading and proliferation [109]. There is also no concern about the transmission of pathogens [110]. The unique amino acid sequence (i.e., glycine, proline and hydroxyproline)-RGD is valuable because it promotes cell adhesion and differentiation. Low antigenicity, high solubility, biodegradability and biocompatibility make gelatin suitable for hard and soft tissue applications [111].

Gelatin possesses the unique property of transitioning between colloidal and gel states, a characteristic derived from the denaturation of its original polymer. This denaturation disrupts the natural triple helix configuration of collagen fibers, allowing partial recovery upon cooling. Gelation occurs between 35 and 40 °C, preserving a colloidal solution; below this range, reconstruction of triple helices leads to gel formation [112]. The chemical structure comprises hydrophilic polypeptide chains that are slightly or strongly cross-linked. Because of this, gelatine can be dissolved in water using different configurations, such as hydrogels, fibers, films or scaffolds [113].

The incorporation of bioactive glass into the gelatin matrix primarily improves mechanical stability due to the BG particles acting as a reinforcement, respectively, increasing the degradation rate of the resulting material in the physiological environment by its hydrophilicity and the release of BG ions into the aqueous environment accelerating the degradation kinetics [114].

The use of gelatin as a copolymer in bioactive glass-based injectable materials improves bioactivity and injectability. A limitation of these materials is their temperature sensitivity, which implies low stability when injected into the body [115]. For this, a third component can be used as a suitable crosslinker, such as polyethylene glycol, β-glycerophosphate, hydroxyl propyl methylcellulose, glutaraldehyde [116,117]. In addition, gelation time decreases and mechanical strength increases when gelatin is used as a copolymer in chitosan–bioglass-based hydrogel [117].

### 3.5. Pectin

Pectin is a linear polysaccharide polymer produced from the primary cell walls of soil plants, such as sunflower heads, citrus fruits and apple peels, by chemical or enzymatic methods [118,119]. It has recently been exploited for extracellular matrix repopulation in tissue regeneration; it is easily degradable, suggesting ideal biocompatibility and biosecurity [120,121].

Chemically, pectin consists of partially esterified/amidated galacturonic acid residues linked by linear 1,4-glycosidic bonds with varying degrees of methylation of carboxylic acid residues, which influence its gelling properties. Depending on the degree of esterification (DE), pectins can be classified as low methoxyl (DE < 50%) or high methoxyl (DE > 50%). Low methoxyl pectins are able to form gels in the presence of divalent ions, such as Ca^2+^, over a wider range of pH values. Methoxy groups can be converted to amide groups, which change the properties of the gel, requiring less calcium for hydrogel formation and being less prone to precipitate at high Ca^2+^ concentrations [121,122].

Pectin is used in various pharmaceutical and biomedical applications, particularly in the treatment of cancer, due to its versatility. However, its low mechanical strength and rapid degradation rate limit its use as a drug delivery device and as a tissue engineering scaffold. One solution to combat these problems is to combine pectin with other biopolymers and/or inorganic compounds [123].

Regarding hydrogels, one approach to induce cross-linking is internal gelation, using inorganic agents such as CaCO_3_, hydroxyapatite and bioactive glass in particulate form. These are dispersed in the polymer solution and act as release vehicles for the slow release of Ca^2+^ ions that cross-link the polymer solution, thus creating homogeneous hydrogel networks [122,124]. Calcium ions are rapidly dislodged from the egg-box structure of pectin by other counterions (such as Na^+^), leading to the formation of soluble pectin from the insoluble gel. Pectin in gel form has become attractive due to its mild solubility modulation compared to other natural polymers such as alginates. In addition, gels can promote the nucleation of a mineral phase on pectin microspheres, with the formation of a biomimetic construct, which better imitates the natural architecture of bone [119].

### 3.6. Hyaluronic Acid

Hyaluronic acid is a major component of the extracellular matrix of the joints and skin and is synthesized at the cell surface [125]. In terms of chemical structure, it is a polysaccharide with a high molecular mass (1000 and 8000 kDa) and consists of repeating units of N-acetyl-D-glucosamine and D-glucuronic acid [77]. The discovery of hyaluronic acid and the methods to obtain it have generated significant interest in the biomedical applications of this biopolymer [109]. It is now produced by microbial fermentation, for example, by *Streptococcus zooepidemicus* or by extraction from animal tissues such as rooster combs. However, both methods suffer from low yields that increase the cost of the feedstock compared to other natural polymers and raise concerns about the safety of using the biomaterial [126].

Hyaluronic acid has a high renewal rate in the body and is biodegradable, contributing to cell proliferation and migration. Due to these advantages, this polysaccharide has numerous applications, such as drug delivery, tissue engineering and surface modification. Hyaluronic acid is a fluid paste that flows under its own weight [127]. By incorporating inorganic materials such as bioactive glass or calcium phosphate, these composites have been shown to be suitable for injection with biodegradability, intense mineralization and mechanical strength [128]. These properties have made these composite gels suitable for applications in bone tissue engineering [129].

### 3.7. Gellan Gum

One polysaccharide that has begun to receive attention in tissue engineering is gellan gum, produced by bacteria such as *Sphingomonas paucimobilis*. It is a non-toxic polymer, which is available at a low price and avoids regulatory concerns about the use of animal products [130]. In situ gelation and the adaptation of the degree of cross-linking have led to gellan gum being investigated for various regenerative medical applications [131]. However, as with most natural polymers, the major drawback is its low mechanical properties [132].

In terms of chemical composition, native gellan gum is composed of repeating units of glucose, glucuronic acid and rhamnose, and two acyl groups, acetate and glycerate, which are linked to the glucose residue adjacent to glucuronic acid [133]. It consists of around 20% rhamnose, 20% glucuronic acid and 60% glucose [134]. There are two commercially available forms, acetylated and deacetylated, with the most common and available being deacetylated gellan gum. It becomes a hydrogel through a transition from the spiral form present at high temperature to a double helix form at room temperature.

Gelation of the polymer is induced by adding cations to the gellan gum solution and occurs gradually as the solution cools [135]. The nature of the cations used is important, with divalent cations strongly favoring gelation over monovalent cations [136]. The use of hydrazone as a potential cross-linker has proven to be effective, resulting in hydrogels with improved properties that can be exploited in soft tissue engineering, with the material having a stiffness similar to that of soft tissues [137]. Furthermore, if hydrogels are freeze-dried and specifically rehydrated, they can become sponge-like hydrogels that will exhibit different characteristics.

The formulation of injectable materials based on gellan gum and bioactive glass particles has the advantage of cross-linking in the presence of divalent Ca^2+^ cations, which function as potential ionic cross-linkers in the formation of gellan gum hydrogel [138]. It has been shown that the use of a methacrylated gellan gum and collagen-based material with bioglass particles can induce an autonomous osteogenic differentiation response of human adipose-derived stem cells [139].

## 4. Bioactive Glass-Based Formulations in Tissue Engineering

The incapacity of the human body to autonomously repair bone defects has necessitated the appearance of conventional therapeutic methodologies. These include techniques such as distraction osteogenesis, bone transport, various autologous and allogeneic bone grafts, along with growth factors [140,141,142]. These approaches have become standard practice in clinical settings, playing a pivotal role in inducing or enhancing bone regeneration [143,144]. In this context, novel approaches have been made to advance bioactive glasses, which are characterized by modifications in composition, the formation of composites and methods of implantation. These innovative materials are beginning to make valuable contributions to the biomedical field, especially in the domain of orthopedic applications [145,146].

The incorporation of bioactive glass under various compositions has yielded innovative formulations, offering versatile solutions in the form of both cements and hydrogels. This chapter explores formulations where bioactive glass plays a pivotal role, serving as a key component in inorganic pastes and organic hydrogels. These formulations represent a convergence of inorganic and organic matrices, presenting a novel approach to tissue repair and regeneration.

Bone cement, as a type of bone filler, finds extensive application in diverse orthopedic and dental implant fixations. The optimal bone cement should serve as an adhesive substrate for osteoprogenitor cells to deposit bone matrix and progressively mineralize into bone. To achieve this, the cement should undergo slow absorption, enabling the infiltration and growth of newly formed bone into the material [147]. Among the most employed bioglass cements for bone regeneration therapies is calcium phosphate, which is appreciated for its biological attributes such as biocompatibility, bioactivity and osteoconductive properties [148]. Table 1 illustrates the broad applications of cement materials employed in conjunction with bioactive glass.

Hydrogels based on bioactive glass find application in tissue engineering due to their flexibility and compatibility with the body. The characteristics of an optimal injectable hydrogel involve considerations of mechanical strength, viscoelasticity and biocompatibility. Research indicates that collagen and chitosan are the predominant materials combined with bioactive glass, likely due to their exceptional properties in mimicking the natural extracellular matrix and facilitating tissue regeneration (Table 2).

Based on their composition, three classes of bioactive glasses are available: silicate, borosilicate and phosphate-based BG; however, limited studies are available, especially limited comparative studies.

Silicate-based bioactive materials, such as bioactive glass, have demonstrated an excellent ability to form bone-like carbonated hydroxyapatite [198]. In vitro and in vivo experiments [182,199] have shown that ion release from silicate-based BG activates macrophages to the M2 phenotype, stimulating anti-inflammatory and angiogenic growth factors. Additionally, in vitro studies revealed that BG promotes faster migration of fibroblasts and endothelial cells, increasing fibroblast production of proteins and growth factors [200]. Specifically, silica ions stimulate osteoprogenitor cells at the genetic level, enhancing osteoblast migration, adhesion and proliferation at defect sites [201]. However, the lower biological activity of these glasses is attributed to their interconnected linkages and high silica content, which prevents the active components from easily leaving the surface [202].

Borosilicate is the second most studied type of bioactive glass after silicate-based BG. Numerous studies have demonstrated that the boron content in BG significantly impacts its mechanical and structural properties [1,203,204]. In borosilicate bioactive glass, the presence of boron reduces reactivity and slows down the formation of apatite, which is essential for bone regeneration. However, boron’s higher solubility results in faster dissolution of the glass in bodily fluids [205,206]. Specific types of borate-based bioactive glass exhibit exceptionally high elastic modulus and compressive strength. The high concentration of boron released at the implantation site from borosilicate glasses slows the degradation of biomaterials and increases the pH of the growth medium [201]. In vitro tests have demonstrated that borosilicate glasses exhibit bioactive behavior [203,207]. In vivo studies on volumetric muscle loss, as reported in Jia’s paper [208], have shown that borosilicate glass possesses a desirable boron release profile. This release profile is crucial in promoting angiogenesis and the proliferation of stem cells.

Phosphate-based glasses, with P_2_O_5_ as the network-forming oxide, offer excellent biocompatibility and bioactivity across various biomedical applications [209]. They can be enhanced with therapeutic ions, even if they are more prone to collapsing and crystallization during mesoporous structure formation [210]. In vitro and in vivo studies have demonstrated that phosphate-based BG are bioresorbable, interacting with physiological fluids to produce the desired biological response and facilitate the regeneration of outgrowing axons [211]. Their composition, resembling bone and teeth more closely than silicate-based glasses, enables mesoporous phosphate-based BGs to induce hydroxycarbonate apatite formation on their surface [212]. Nonetheless, phosphate-based glasses encounter challenges in long-term stability compared to silicate glasses due to lower chemical durability [212,213]. In a study conducted by McLaren [214], it was demonstrated that phosphate-based glass microspheres are biocompatible and promote new bone tissue formation and osteointegration, making them suitable candidates for tissue regeneration applications.

Recent approaches are related to the development of composite materials combining calcium phosphate (especially β-TCP) and bioactive glasses. The resulting composite powders, as reported by Nakanishi et al. [215], for instance, have good porosity and promote a good proliferation of the osteoblast cells, while Sujon et al. [216] proved that the composite obtained between hydroxyapatite and 45S5 BG presented enhanced cell adhesion and proliferation, and induce good hydroxyapatite forming ability. These results can be a consequence of the coexistence of the individual BG and CPC phases, which are beneficial at least for several properties, especially biological properties. A more complex structure is reported by Park et al. [217] based on CaO-SiO_2_-P_2_O_5_-B_2_O_3_ bioactive glass ceramic or PEK cage filled with hydroxyapatite/β-TCP.

In recent years, the incorporation of various biologically active ions into silicate, phosphate and borosilicate bioactive glass systems has gained popularity. These additions aim to enhance functional properties such as osteogenesis, angiogenesis, bioactivity and antibacterial effects. For example, boron-based bioactive glass increases tissue regeneration when doped with elements like magnesium, zinc and strontium [203,218]. Phosphate-based bioactive glass doped with silver has demonstrated improved antimicrobial properties and better dissolution in vitro due to the phosphate content [219]. Additionally, the doping with strontium enhances the bioactivity and mechanical properties of phosphate glass materials [220]. Silicate glasses doped with zinc, silver or gallium have shown antibacterial activity against bone-specific bacterial strains [221,222,223,224], while copper-doped BG also promoted angiogenesis in vivo studies. Ozel et al. [225] demonstrated that strontium and zinc co-doped bioactive glass has a higher bioactivity, as indicated by the increased crystallinity of hydroxyapatite formations compared to undoped bioactive glasses [224]. Numerous papers explore these BGs and the role of ion-doping with the commonly used biologically active ions, most of the conclusions being similar to that obtained in the case of doping hydroxyapatite, but the higher bioactivity can lead to a higher level of these ions [202,226,227,228].

**Figure 3:**
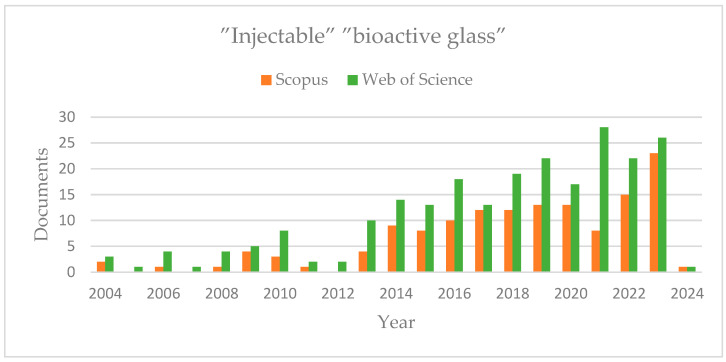
Number of articles on injectable bioactive glass (2004–present).

A systematic exploration of Scopus and Web of Science databases, focusing on articles incorporating the terms “bioactive glass” and “injectable,” was conducted, covering data from 2004 onward. The inquiry generated 140 results in Scopus and 233 in Web of Science. When the search is refined using these keywords, insights into the increasing interest in bioactive glass across diverse fields are gained. The chart below illustrates the number of articles found each year, indicating a significant spike in research on injectable bioactive glass, especially from 2020 onward (Figure 3).

## 5. Conclusions and Future Directions

The exploration of appropriate biomaterials for bone regeneration stands as a complex challenge in biomedical research. The investigation into bioactive glasses emerges as a captivating research domain with encouraging outcomes. Over the past few decades, it has undergone comprehensive examination, addressing various challenges in the field of bone tissue engineering. L. Hench’s discoveries have served as a catalyst for numerous studies exploring various aspects of bioactive glass. While the majority still focus on the original 45S5 composition and implantation, a wealth of reports in the literature strongly suggest that novel bioactive glass formulations and delivery methods present fewer limitations than the original counterpart [71,179,229]. Despite the promising results achieved thus far, additional research is imperative to solidify and comprehend all the complexities linked with bioglass. This includes a detailed examination of its specific compositions and an exploration of the impacts of long-term degradation under both in vivo and in vitro conditions, crucial for its eventual clinical application in bone regeneration. Current works are trying to develop composites based on BG and different CPCs, as well as polymers, in order to tune the final characteristics of these materials further.

## Figures and Tables

**Table 1:** Applications of bioactive glass-based cements.

Composition	Glass Composition (Molar Ratio)	Applications	References
Acrylic Bone Cements	58SiO_2_-36CaO-6P_2_O_5_	Drug delivery system	[27]
	45SiO_2_-24.5Na_2_O-24.5CaO-6P_2_O_5_	Denture base materials	[28]
	45SiO_2_-24.5Na_2_O-24.5CaO-6P_2_O_5_	Coatings	[29]
	5.5 Na_2_O-11.1 K_2_O-4.6MgO-18.5CaO-56.6B_2_O_3_-3.7P_2_O_5_	Bone regeneration	[149]
	45SiO_2_-24.5Na_2_O-24.5CaO-6P_2_O_5_	Bone regeneration	[150]
	24.5 SiO_2_-38CaO-12.7P_2_O_5_-24.8MgO	Bone regeneration	[151]
	Ag doped 48SiO_2_-18Na_2_O-30CaO-3P_2_O_5_-0.43B_2_O_3_-0.57Al_2_O_3_	Temporary prostheses	[152]
Calcium Sulphate Cements	60SiO2-35CaO-5P_2_O_5_	Tissue graft	[39]
	SiO_2_-CaO-P_2_O_5_-B_2_O_3_-MgO	Polymorphic bone defect repair	[153]
	80SiO2-15CaO-5P_2_O_5_	Bone repair and drug release	[154]
	45SiO_2_-24.5Na_2_O-24.5CaO-6P_2_O_5_	Bone substitute	[155]
	45SiO_2_-30CaO-5P_2_O_5_-2B_2_O_3_-15CaCl_2_-3MgO	Orthopaedics-vertebroplasty and kyphoplasty	[156]
Calcium Phosphate Cements	45SiO_2_-24.5Na_2_O-24.5CaO-6P_2_O_5_	Cell proliferation and osteogenic differentiation	[157]
	45SiO_2_-24.5Na_2_O-24.5CaO-6P_2_O_5_	Bone regeneration	[158,159]
	30.67CaO-43.14P_2_O_5_-9.42Na_2_O-14.32K_2_O-2.45MgO	Bone implant	[160]

**Table 2:** Applications of bioactive glass-based hydrogels.

Composition	Glass Composition (Molar Ratio)	Applications	References
Alginate	45SiO_2_-24.5Na_2_O-24.5CaO-6P_2_O_5_	Therapeutic angiogenesis	[161]
	SiO_2_-CaO-P_2_O_5_	Scaffolds in bone tissue engineering	[162,163]
	80SiO_2_-15CaO-5P_2_O_5_	Scaffolds in bone tissue engineering	[164]
	45SiO_2_-24.5Na_2_O-24.5CaO-6P_2_O_5_	Skin repair-promoting biomaterials	[165]
	49.46SiO_2_-6.6Na_2_O-27.27CaO-1.07P_2_O_5_-3SrO-6.6K_2_O-3MgO-3ZnO	Scaffolds in bone tissue engineering	[166]
Chitosan	60SiO_2_-36CaO-4P_2_O_5_	Bone regeneration	[167]
	Ag-60SiO_2_-36CaO-4P_2_O_5_	Dental pulp repair	[168,169]
	64SiO_2_-31CaO-5P_2_O_5_	Scaffolds in bone tissue engineering	[170]
	Sr-45SiO_2_-24.5Na_2_O-24.5CaO-6P_2_O_5_	Repairing large bone injuries	[171]
	85SiO_2_-10CaO-5P_2_O_5_	Scaffolds in bone tissue engineering	[104,172]
	55SiO_2_-40CaO-5P_2_O_5_	Alveolar bone tissue engineering	[173]
	58SiO_2_-33CaO-9P_2_O_5_	Bone regeneration	[174]
Collagen	45SiO_2_-24.5Na_2_O-24.5CaO-6P_2_O_5_	Scaffolds in bone tissue engineering	[175]
	85SiO_2_-15CaO	Stem cell culture for bone tissue engineering	[176]
	53SiO_2_-23Na_2_O-20CaO-4P_2_O_5_	Bone tissue engineering	[177]
	58SiO_2_-33CaO-9P_2_O_5_	Scaffolds in bone tissue engineering	[178]
Gelatin	55SiO_2_-24CaO-6P_2_O_5_-15B_2_O_3_	Scaffolds in bone tissue engineering	[179]
	64SiO_2_-5P_2_O_5_-26CaO-5MgO	Scaffolds for nerve regeneration	[180]
	45SiO_2_-24.5Na_2_O-24.5CaO-6P_2_O_5_	Hydrogel in hard tissue engineering	[181]
	64SiO_2_-27CaO-4MgO-5P_2_O_5_	Small bone defect	[89]
	Ag-58SiO_2_-33CaO-9P_2_O_5_	Bone tissue engineering	[182]
	54.2SiO_2_-35CaO-10.8P_2_O_5_	Pulp regeneration	[183]
	45SiO_2_-24.5Na_2_O-24.5CaO-6P_2_O_5_	Drug delivery as 3D sponge-like scaffolds	[184]
	SiO_2_-B_2_O_3_-CaO-K_2_O-MgO-Na_2_O-P_2_O_5_	Bone tissue regeneration	[185]
Pectin	40SiO_2_-54CaO-6P_2_O_5_	Injectable hydrogel in bone tissue engineering	[122]
	45SiO_2_-24.5Na_2_O-24.5CaO-6P_2_O_5_	Soft and hard tissue engineering	[186]
	45SiO_2_-24.5Na_2_O-24.5CaO-6P_2_O_5_	Fiber construct in bone regeneration	[187]
Hyaluronic acid	60SiO_2_-36CaO-4P_2_O_5_	Critical-size bone defect repair	[188]
	SiO_2_-CaO-P_2_O_5_	Scaffolds or coating in tissue engineering	[189]
	47.5SiO_2_-2.5P_2_O_5_-20CaO-20MgO-10Na_2_O-10K_2_O	Scaffolds in bone tissue engineering	[190]
	SiO_2_-Na_2_O-K_2_O-CaO-MgO-P_2_O_5_	Soft tissue engineering	[191]
	58SiO_2_-33CaO-9P_2_O_5_	Bone tissue engineering	[192]
	50SiO_2_-45CaO-5P_2_O_5_	Coating in orthopaedic implants	[193]
	45SiO_2_-24.5Na_2_O-24.5CaO-6P_2_O_5_	Injectable bone substitute	[194]
Gellan gum	54SiO_2_-40CaO-6P_2_O_5_	Injectable bone substitute	[195]
	70SiO_2_-30CaO	Bone tissue engineering	[196]
	66SiO_2_-10Na_2_O-22CaO-2P_2_O_5_ 70SiO_2_-30CaO	Scaffolds in bone tissue engineering	[132]
	43.7SiO_2_-10.9B_2_O_3_-22.1CaO-7.9K_2_O-7.7MgO-6.0Na_2_O-1.7P_2_O_5_	Bone tissue engineering	[197]
